# The Causation of Chloroform Syncope

**Published:** 1899-02

**Authors:** 


### SELECTIONS.

### The Causation of Chloroform Syncope.

Leonard Hill combats the doctrine of the Hyderabad Commission and of Lawrie, that chloroform kills by paralyzing the respiratory centre, and reaches the following conclusions:

1. Chloroform produces a primary failure of the circulating mechanism and a secondary failure of the respiratory centre. The respiratory centre fails to act, not only because it is damaged by the drug, but because of the anemia of the spinal bulb produced by the fall of arterial tension. This is proved by the fact that the action of the respiratory centre can be renewed by raising the arterial tension. The depth of anesthesia depends, as does the paralysis of the respiratory centre, on the primary fall of the arterial tension.

2. Chloroform, more than any other known agent, rapidly abolishes the vascular mechanisms which compensate for the hydrostatic effect of gravity.

3. Chloroform abolishes these mechanisms by paralyzing the splanchnic vaso-motor tone and by weakening the action of the respiratory pump. When these mechanisms are totally abolished the circulation is impossible, if the subject be in a feet-down position.

4. Chloroform also produces paralytic dilitation of the heart. It acts directly like amyl-nitrate, on the musculature of the whole vascular system.

5. There are two forms of chloroform syncope: (*a*) During primary anesthetizations. The patient struggles, holds his breath, raises the intra-thoracic pressure, congests his venous system, lowers his arterial tension, and finally, takes deep inspirations and surcharges his lungs with chloroform. In the first stages the left heart becomes impoverished; in the second stage it is suddenly filled with blood. This is drawn from the lungs and is full of chloroform. The chloroform passes into the coronary arteries and the heart is thrown into paralytic dilatation. Respiration and the pulse either cease simultaneously or the pulse before respiration. (*b*) During prolonged anesthetization this arises from gradually giving chloroform to too great an extent. The arterial pressure falls lower and lower, and secondarily, the respiration ceases because of the anemia of the spinal bulb. The heart is not, in this case, paralyzed by chloroform, because the drug is taken in gradually by the shallow respirations and distributed slowly by the feeble circulation.

6. Artificial respiration and the assumption of the horizontal position, if applied in time, will always resuscitate a patient from the second form of syncope.

7. Artificial respiration, established with the patient in the horizontal position, is also the treatment indicated in the first form of syncope; the heart should be rhythmically compressed by squeezing the thorax. If this does not quickly renew the pulse, the patient should be put into the vertical feet-down posture. The dilated right heart is thereby easily and completely emptied of blood. Artificial respiration is maintained during this maneuver and the patient is brought once more into the horizontal posture. By rhythmic compression of the chest an efficient circulation is maintained through the coronary arteries by first emptying and then filling the heart, a fresh supply of blood is brought into the organ. If this does not prove successful on the first trial it can be repeated.

8. Inversion—that is, placing the subject in the feet-up position—or compression of the abdomen will increase the paralytic dilatation of the heart. In this kind of syncope both these forms of treatment are worse than useless.

9. In the condition of shock or emotional fear the compensatory mechanism for the effect of gravity is almost abolished and chloroform may easily be the last straw to completely paralyze the circulation.

10. Vagus inhibition of the heart is of no importance as an agent in the production of chloroform syncope.

11. Ether is, in every respect, a far safer anesthetic than chloroform. According to Ringer's experiments on the heart, ether is fifty times less dangerous than chloroform.

12. With the practical conclusion of the Hyderabad Commission, that the chloroform-inhaler should be removed during the struggling of the patient or when the respiration is of irregular depth, he absolutely agrees, but he considers their interpretation of their own experiments and tracings concerning the origin of chloroform syncope to be mistaken.

Not only the work of all physiologists, but also the tracings of the Commission, when rightly interpreted prove that paralysis of the circulatory mechanism and not of the respiratory centre, is to be dreaded by the anesthetist.—*Louisville Med. Monthly.*

